# On the use of a continuous metabolic syndrome score in pediatric research

**DOI:** 10.1186/1475-2840-7-17

**Published:** 2008-06-05

**Authors:** Joey C Eisenmann

**Affiliations:** 1 Department of Kinesiology, Michigan State University, East Lansing, USA

## Abstract

**Background:**

The constellation of elevated levels of abdominal adiposity, blood pressure, glucose, and triglycerides and lowered high-density lipoprotein-cholesterol has been termed the metabolic syndrome. Given the current pediatric obesity epidemic, it is perhaps not surprising that recent reports suggest the emergence of the metabolic syndrome during childhood and adolescence. The aim of this paper is to provide an overview of the derivation and utility of the continuous metabolic syndrome score in pediatric epidemiologic research.

**Methods/Design:**

Data were generated from published papers related to the topic.

**Conclusion:**

Although there is no universal definition in children or adolescence, recent estimates indicate that approximately 2–10% of youth possess the metabolic syndrome phenotype. Since there is no clear definition and the prevalence rate is relatively low, several authors have derived a continuous score representing a composite risk factor index (i.e., the metabolic syndrome score). This paper provides an overview of the derivation and utility of the continuous metabolic syndrome score in pediatric epidemiological research.

## Background

The constellation of adverse cardiovascular disease (CVD) and metabolic risk factors, including elevated abdominal obesity, blood pressure, glucose, and triglycerides (TG) and lowered high-density lipoprotein-cholesterol (HDL-C), has been termed the metabolic syndrome [1]. Based on the National Cholesterol Education Program Adult Treatment Program III (ATP II) criteria [1] and the International Diabetes Foundation criteria [2] approximately 35% and 39%, respectively, of U.S. adults possess the metabolic syndrome [3]. In terms of health outcomes, the metabolic syndrome is associated with an increased risk of all-cause mortality, CVD morbidity and mortality, and diabetes [4].

Given the current pediatric obesity epidemic [5], it is perhaps not surprising that recent reports indicate the emergence of the metabolic syndrome during childhood and adolescence [6-10]. Data from the United States (U.S.) National Health and Nutrition Examination Survey (NHANES) III (1988–1994) showed that the prevalence rate of the metabolic syndrome was 4% in 12–19 yr old adolescents [10]. The prevalence rate increased to 6% in NHANES 1999–2000 [9]. Based on current estimates, > 2 million U.S. adolescents have the metabolic syndrome phenotype [9]. Another paper using the same data (i.e., NHANES III) but different criteria showed that the prevalence rate was nearly 10% [11]. The varying prevalence rates between studies reflects the lack of a universal definition and makes it difficult to draw conclusions. This issue is similar in the adult literature [12]. Prevalence rates from representative samples in other countries are limited. The metabolic syndrome in children and adolescents has been reported to be 6.5% in northern Mexico[13], 9% in Korea [14], 2% in Turkey [15], and 10% in Quebec, Canada [16]. Among obese children and adolescents the prevalence approximates 30–50% [10,17,18].

Since there is no universal definition of the metabolic syndrome in children or adolescence and the prevalence rate is relatively low, several authors [19-28] have derived a continuous score representing a composite CVD risk factor profile or index (i.e., the metabolic syndrome score). The purpose of this paper is to provide an overview of the derivation and utility of the continuous metabolic syndrome score in pediatric epidemiologic research.

## Rationale for a continuous metabolic syndrome score

The rationale for creating a continuous metabolic syndrome score stems mainly from the fact that there is no clear definition of the metabolic syndrome in children or adolescence and the prevalence rate is relatively low. A low prevalence rate requires a large sample size in order to conduct association studies. For example, in a random sample of 500 U.S. adolescents approximately 15–50 subjects would possess the metabolic syndrome based on current estimated prevalence rates. Thus, the ability to show links between exposures (e.g., physical activity, diet, etc.) and the dichotomous outcome (i.e., metabolic syndrome) using logistic regression would limit the power to detect an association [29].

As mentioned, several authors have derived a continuous score to represent the clustering of components of the metabolic syndrome (Table 1). The inclusion of these key components (i.e., glucose, lipids, blood pressure, and adiposity) is supported by the results of factor analysis in children and adolescents [16,30-32] which shows the underlying patterns or structure among variables showing high degrees of inter-correlation. Thus, there are common variables that can be used to calculate a metabolic syndrome score. It has also been argued that a continuous metabolic syndrome score is statistically more sensitive and less error prone by comparison to the dichotomous approach [20,29]. In the recent joint statement by the American Diabetes Association and the European Association for the Study of Diabetes [33], it was recommended that one area of necessary research was the definition of the metabolic syndrome based on continuous variables in a multivariate score system.

**Table 1:** Summary of approaches used to calculate the continuous metabolic syndrome score in pediatric epidemiological research.

**Study**	**Obesity**	**Lipids**	**Glucose or Insulin**	**Blood Pressure**	**Other**	**Statistical approach**
Bogolusa Heart Study [21]	-	TC:HDL	Insulin	SBP		Sum of the individual rankings by age-, sex-, and race-specific levels
Young Danes [28]	Skinfolds	TC, HDL-C, TG	-	SBP and DBP	smoking	Upper centiles
Cardiovascular Risk in Young Finns [22]	-	TC and HDL-C	-	DBP		Upper tertile
European Youth Heart Study [20]	Skinfolds	HDL-C and TG	Glucose and insulin	Average of SBP and DBP		Sum of six Z scores divided by 6
European Youth Heart Study [19]	Skinfolds	TG and TC:HDL-C	HOMA	SBP	Aerobic fitness	Sum of Z scores
Corpus Christi Child Heart Study [23]	BMI	HDL-C and TG	Insulin	SBP		2 approaches: 1) sum of Z scores, and 2) principal components analysis
Quebec Family Study [24]	Skinfolds	HDL-C, TG, and TC:HDL-C	Glucose	MAP		principal component analysis
Aerobic Center Longitudinal Study [27]	WC	HDL-C and TG	Glucose	MAP		Sum of age-standardized residuals
Australian Health and Fitness Study [34]	WC	HDL-C and TG	-	MAP		Sum of age-standardized residuals
Physical Activity across the Curriculum [38]	WC	HDL-C and TG	HOMA	MAP		Sum of age-, sex-, and race-standardized residuals

BMI, body mass index; WC, waist circumference; TC, total cholesterol; HDL-C, high density lipoprotein-cholesterol; TG, triglycerides; HOMA, homeostasis model assessment; SBP, systolic blood pressure; DBP, diastolic blood pressure; MAP, mean arterial pressure.

## An overview of methods used to derive the metabolic syndrome score

Several large-scale epidemiological studies that either focus on cardiovascular health or include CVD risk factors in children and adolescents have used various approaches to calculate a metabolic syndrome score (Table 1). As shown in Table 1, the variables included in the score and statistical approaches used to derive the score vary considerably. Indicators of obesity range from the body mass index (BMI) to skinfolds to waist circumference (WC), whereas others do not include a measure of adiposity. The same is true for the other components of the metabolic syndrome. To my knowledge, Eisenmann and colleagues are the only ones to use indicators aligned with the adult criteria. More specifically, WC, HDL-C, TG, mean arterial pressure (MAP) and some indicator of abnormal glucose metabolism are used. Besides differences in the variables included in the score, the statistical approaches used to calculate the score also vary considerably. Previous procedures include principal component analysis [23,24], Z scores [19,20,23,25-27,34], and centile rankings [21,22]. In addition, a recent study proposed a categorical score referred to as the Metabolic Individual Risk-factor And Clustering Estimation (MIRACLE) score based on family history (early cardiovascular disease, type 2 diabetes, and hypertension), individual history (small for gestational age and ethnic origin), clinical features (BMI, waist circumference > 90th percentile and blood pressure > 95th percentile) and metabolic abnormalities (glucose intolerance or type 2 diabetes) [35].

## An example of the calculation of the metabolic syndrome score using the Z score method

In the past few years, we have used the Z score approach to calculate the metabolic syndrome score. In this section, some general considerations and the specific methodology of this approach are highlighted.

### Step 1: Choice of variables to be included in the metabolic syndrome score

The first step is to choose the variables to be represented in the score. As mentioned, several variables have been used in the calculation of the metabolic syndrome score. In the opinion of this author, the variables should reflect the adult metabolic syndrome criteria as discussed above. However, some data sets may lack some of the variables consistent with the adult criteria. For example, fasting blood glucose or WC may not have been measured in some studies. In the case where WC is not available, the BMI or skinfold thickness has been used. Another issue we have faced is deciding upon which blood pressure variable should be used. Instead of using both systolic and diastolic blood pressure (or the average – e.g., Brage et al.), we have chosen MAP to represent blood pressure. Likewise, the inclusion of glucose and/or insulin is a challenge. Since fasting blood glucose is typically normal in youth and even in overweight youth [10], other parameters of insulin resistance should be used. In recent analyses, we and the European Youth Heart Study [19] chose to represent insulin resistance as the homeostasis assessment model of insulin resistance (HOMA)[36].

### Step 2: Choice of variables included in the standardization process

In our previous studies, the individual risk factors have been regressed onto age to account for any age-related differences in the risk factors. Unfortunately, we have not been able to account for biological maturity status. It is recommended to include a biological maturity indicator in this procedure since maturity status also influences the individual risk factors. In addition, other demographic variables, such as sex, ethnicity, and socio-economic status, can be considered here. Again, a decision needs to be made regarding which variables to include in this step. When sufficient numbers of boys and girls or ethnic groups are available, separate analyses can be conducted for sex and ethnicity. On the other hand, if the sample size for these groups is insufficient for group specific analyses, the individual risk factors can be standardized by age, sex, and ethnicity.

### Step 3: Calculation of individual Z scores and the metabolic syndrome score

Once the decision has been made as to which variables will be included in the score and which variables will be used to standardize the score, the statistical procedures can then be performed. The initial statistical procedure is to standardize the individual metabolic syndrome variables (e.g., WC, MAP, HOMA, HDL-C, and TG) by regressing them onto the selected demographic variables (e.g., age, sex, ethnicity, etc.). Once each metabolic syndrome variable has been regressed onto the independent variables, the standardized residual is saved (e.g., Z_WC, etc.). Since the standardized HDL-C is inversely related to metabolic risk it is multiplied by -1. The standardized residuals (Z-scores) for the individual risk factors (WC, MAP, HDL-C, TG, and HOMA) are summed to create the metabolic syndrome score. A higher score is indicative of a less favorable metabolic syndrome profile.

**Figure 1:**
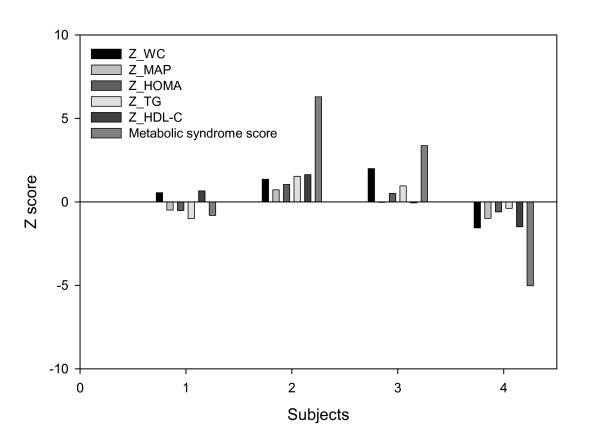
**Illustrative example of the age-, sex-, and race-standardized Z score used in the derivation of the metabolic syndrome score.** See table 2 for abbreviations and measured values.

For a specific example, Table 2 shows demographic information, values for individual risk factors and the Z scores for 4 girls participating in a study that included 193 girls and 182 boys ages 7 to 9 years of age [8]. The individual Z scores and the metabolic syndrome score are also illustrated in Figure 1. The metabolic syndrome score was derived by calculating age-, sex-, and race-specific Z scores for WC, MAP, HOMA, HDL-C, and TG and summing the individual Z scores as described above. The highest metabolic syndrome scores are in subjects 3 and 4, both of whom were obese. Subject 2 displayed positive Z scores for each risk factor. Subject 1 displayed the lowest metabolic syndrome score and had negative Z scores for each risk factor. It should be noted that a subject could possess both positive and negative Z scores for various risk factors (Subject 1).

**Table 2:** Example of data and age-, sex-, and race-standardized Z score used in the derivation of the metabolic syndrome score.

			Measured values					Standardized residuals (Z scores)					
Subject	Age	Race	WC	MAP	HOMA	TG	HDL	Z_WC	Z_MAP	Z_HOMA	Z_TG	Z_HDL	MetS Score

1	8	White	63.8	76.0	0.53	48	47	0.55	-0.49	-0.52	-1.01	0.66	-0.81
2	8	White	70.5	85.3	3.33	119	36	1.36	0.72	1.04	1.53	1.63	6.29
3	8	Hispanic	78.5	80.0	3.32	104	58	1.99	-0.03	0.51	0.96	-0.06	3.37
4	7	Black	45.8	72.0	0.56	62	71	-1.56	-0.99	-0.60	-0.39	-1.49	-5.02

*Mean	7.7		59.3	79.7	1.58	75.5	54.4						
*SD	0.6		8.7	7.8	2.06	26.9	11.0						

WC, waist circumference (cm); MAP, mean arterial pressure (mm Hg); HOMA, homeostasis assessment model of insulin resistance; TG, triglyerides (mg/dl); HDL, high density lipoprotein-cholesterol (mg/dl); Z, z score; MetS score, metabolic syndrome score.

*Values indicate sample mean and standard deviation (SD) (n = 193 girls).

#### Limitations of the metabolic syndrome score

A major limitation to the metabolic syndrome score presented in previous studies is that it is sample specific. Therefore, the mean metabolic syndrome score derived in one study cannot be compared to other studies unless the demographic characteristics, distribution of data, and the measures of central tendency and variability are similar in the two samples. The other limitations of previous research have already been mentioned and include the use of different variables and statistical procedures. It is also important to note that most previous studies have not taken into consideration the age- or maturity-related variation in metabolic syndrome risk factors. Finally, the weighting of each individual variable to the final score is considered equal in the Z score approach. In contrast, factor analysis and principal components analysis calculate the loadings of each variable independently.

There is considerable need to develop a universal definition of the pediatric metabolic syndrome [37]. In an excellent review, Cruz and Goran [37] suggested that the same risk factor variables that are used in the adult definition also be used in children so that the definition is consistent across the lifespan for purposes of tracking. In addition, if epidemiological studies are to use the continuous metabolic syndrome score, a standardized method of calculating the score may prove beneficial for comparing studies. An alternative approach to the sample-specific Z score approach highlighted here may be to compare individual risk factor value to the population median (i.e., 85^th ^percentile of age- and sex-specific reference values) or clinical cut-points.

## Utility of the metabolic syndrome score in pediatric epidemiological research

Several authors have shown significant differences or associations between various exposures and the metabolic syndrome score. For example, results from the Corpus Christi Child Heart Study indicated higher metabolic syndrome scores in Mexican-American boys and girls compared to White boys and girls [23]. Recent studies also show that habitual physical activity and aerobic fitness are inversely associated with the metabolic syndrome score [19,20,25-27,34]. Eisenmann and colleagues [25,26,34,38] have also shown that aerobic fitness attenuates the metabolic syndrome score among high fat children and adolescents. The metabolic syndrome score has also been shown to track from childhood/adolescence into young adulthood [21,22,24,27]. Other studies show an association between markers of the metabolic syndrome in childhood and type 2 diabetes and cardiovascular disease morbidities in adulthood [39,40]. These latter findings hold importance to the predictive utility of the score on disease outcomes and confirms that the definition of the metabolic syndrome in youth and the calculation of the metabolic syndrome score should include the same variables as those in the adult criteria for the metabolic syndrome [37].

## Summary and recommendations

This paper provides an overview of the derivation and utility of the continuous metabolic syndrome score in pediatric epidemiology. The continuous score is important in epidemiological research since there is no universal definition of the metabolic syndrome for children or adolescents and the prevalence rate is relatively low. The score allows each subject to have a continuous value with lower values indicating a better metabolic syndrome profile and higher values indicating a poorer metabolic syndrome profile compared to the sample studied. Several variables and statistical procedures have been used to derive the metabolic syndrome score in previous research (Table 1). It is recommended that the five key metabolic syndrome variables be used in the calculation of the score in future research. These variables include 1) central obesity (as measured by WC – or BMI and/or skin fold thickness if WC is not available), 2) low HDL-C, 3) elevated TG, 4) elevated BP (systolic and/or diastolic and/or MAP), and 5) abnormal glucose metabolism (impaired fasting glucose, impaired glucose tolerance, and/or HOMA). Furthermore, the individual components should be age-standardized (and maturity-standardized, if available) given the influence of growth and maturation on the development of the metabolic risk factors.

Future research is needed to validate the current methodologies used on deriving the metabolic syndrome score in children and adolescents. Recently, Winjndaele et al [41] showed a continuous metabolic syndrome score derived from principal component analysis was higher in adult subjects with the metabolic syndrome and that the score increased progressively with increasing number of adverse risk factors. Although the metabolic syndrome score tracks from adolescence into adulthood [24,42], association studies using established cohorts that link the metabolic syndrome score during childhood and adolescence with adult-diagnosed metabolic syndrome, type 2 diabetes, atherosclerosis and CVD mortality are required. If a simple and practical yet valid method utilizing criterion-reference standards can be developed, then the metabolic syndrome score could be used conventionally in pediatric epidemiological research, clinical medicine, and public health to better understand the prevention, diagnosis, and treatment of this emerging pediatric condition.

## Competing interests

The author declares that they have no competing interests.

## Authors' contributions

JCE is the sole author of this manuscript.
